# The Impact of Childhood Trauma on Sensory Processing and Connected Motor Planning and Skills: A Scoping Review

**DOI:** 10.1007/s40653-023-00598-y

**Published:** 2023-12-08

**Authors:** Rebecca Matson, Vikki Barnes-Brown, Rachel Stonall

**Affiliations:** School of Health Sciences, University of Liverpool, Liverpool, England

**Keywords:** Trauma, Sensory Integration, Motor Development, Praxis

## Abstract

**Background:**

Traumatic experiences during childhood have been suggested to alter the course of sensory and motor development due to the impact on neural connections within the brain at integral periods. This connection has been alluded to in literature and is discussed anecdotally by practitioners suggesting the impact is commonly seen in practice. Previous scoping reviews in this area have focused solely on the process of sensory modulation without exploring the connection to motor planning.

**Objective:**

This scoping review considers what is known from the existing literature about the impact of childhood trauma on sensory processing and motor skills.

**Method:**

This scoping review followed the JBI methodology for scoping reviews (Peters et al., 2020), searching CINAHL plus, Proquest, Scopus, PsycINFO, EThOS, as well as a search of the reference lists of the articles and citation chaining, to locate both published and unpublished sources. Articles were reviewed by two reviewers independently, with a third reviewer verifying those that met inclusion criteria where there was disagreement.

**Results:**

Six articles were identified that met the inclusion criteria for the study. While all included studies suggested the concurrence of sensory processing and motor difficulties in individuals who have experienced childhood trauma they did not conclusively make the link between the two suggesting an overall low level of evidence. Commonalities were identified in relation to the areas of the brain impacted and the nature of difficulties experienced with some suggestion of this varying according to stage of development and the specific nature of the trauma.

**Conclusions:**

This study suggests emerging evidence in relation to the connection between trauma, sensory processing and motor development but that further empirical research is needed to verify this and inform practice.

## Introduction

### Trauma and Sensory Processing

The impact of trauma on sensory processing is receiving increasing attention, both within and external to the field of occupational therapy. Experiences defined as trauma include abuse, violence, neglect, war and emotional harm (American Psychiatric Association, 2000). Such experiences have been suggested to alter the processing of sensory input due to the long-term impact on neural connections and structures within the brain that are integral in this process (Van der Kolk, 2005; Holland & May-Benson, 2014). Alterations have been found within the brains of childhood trauma survivors in a number of areas that have important roles in sensory integration, that is our ability to process, integrate and organise sensory input both from our own body and the environment to then use our body effectively to interact with the environment around us (Ayres, 1972). Identified areas include the hippocampus, amygdala, thalamus, sensory cortex as well as the pre-frontal cortex (Koomar, 2009; Cozolino, 2010; Engel-Yeger et al., 2013; May-Benson, 2017).

A number of these affected areas have been identified as having an important role in the specific process of sensory modulation, which has been defined as “the capacity to regulate and organize the degree, intensity and nature of responses to sensory input in a graded and adaptive manner” (Miller et al., 2001, p57). As a result, the impact of trauma on sensory modulation has often been the focus of literature in relation to sensory integration and trauma. This impact has been suggested to result from dysregulation of the hypothalamic pituitary adrenal (HPA) axis resulting in fluctuating arousal levels (Lane, 2020). Due to this difficulty with regulation a child who has experienced trauma is often in a state of hyper-vigilance resulting in hyper-responsivity as well as periods of hyporesponsivity to different sensory cues from within their environment (Reeves, 2001; Howard et al., 2020). This impact is considered particularly damaging if the trauma occurs during childhood interrupting the process of sensory development itself which is thought to primarily occur up until age 7 (Van der Kolk, 2005; Holland & May-Benson, 2014; Fraser et al., 2017).

There have been two recent scoping reviews in relation to childhood trauma, sensory modulation dysfunction and intervention (Joseph et al., 2021; Fraser et al., 2017). Joseph et al. (2021) suggested that the inability to regulate sensory input is due to the experience of trauma initially occurring on a somatosensory level and therefore having a significant bodily impact. While their review did not extend beyond sensory modulation this does suggest a need to consider the impact on broader areas of sensory integration due to the centrality of the somatosensory system in development of motor skills. Fraser et al. (2017) suggest that sensory based interventions should form part of the treatment approach with this client group, including sensorimotor based interventions, yet they do not review the evidence for the impact of sensory processing difficulties on motor skills.

### Sensory Integration and Motor Skills

Sensory integration has a developmental focus that sees the senses as foundational to higher level skills including concentration, communication and motor skills (Ayres, 2005; Bundy & Lane, 2020). Difficulties with sensory processing impact on a child’s understanding and exploration of their environment that allows them to develop awareness of their own body, and is a necessary part of motor development (Perry, 2009; Reeves, 2001; Howard et al., 2020). Motor planning and skills are dependent not only on the physical ability to execute the movement but also the cognitive ability to develop an idea of what it is you need to do, to plan how this should be carried out, and the availability of opportunities to develop these skills within our environment, a process described by Ayres (1985) as praxis. For this process to be effective an individual needs an awareness of their body scheme which is informed by integration of sensory information as well as the ability to formulate a motor plan (Cermak & May-Benson, 2020). Trauma has been suggested to impact this process due to a particular effect on the pre-frontal cortex leading to impaired sensory integration and cognition (Harricharan et al., 2021). This may mean that individuals who have experienced trauma struggle with both aspects of this process; that is receiving the necessary feedback from their body but also with the cognitive process of formulating and planning a motor action.

There has also been suggestion of a more indirect connection between trauma and motor skills in that the motor difficulties may occur secondary to the impairment in sensory modulation, rather than as a separate issue. Sensory modulation is an earlier process within the brain than the discrimination of input that is needed to inform motor skills, therefore if this process is disrupted there is reason to consider potential difficulties in other areas of sensory processing and functioning (Holland & May-Benson, 2014; Lane, 2020). If a child is in a constant state of dysregulation this can lead to excessive gating of sensory input that is perceived as a threat by the thalamus (Cozolino, 2010). As a result, insufficient sensory input may progress to the higher levels of the brain to effectively inform skills such as motor planning. This is further compounded by feeling persistently unsafe in and disconnected from their own bodies impacting on body awareness (Van der Kolk, 2015; Perry & Szalavitz, 2017).

### A Sensorimotor Focus in Trauma Treatment

The impact of trauma on motor development has been alluded to in literature and is discussed anecdotally in a number of sources suggesting this connection is commonly seen in clinical practice yet the extent of the evidence for this connection is unclear (Lloyd, 2016; Ogden & Fisher; 2014, Van der Kolk, 2015). However, there has also been a noted shift in clinical practice for trauma towards approaches that use a sensorimotor or bodily-based focus including Sensorimotor Psychotherapy (Ogden et al., 2006) and Eye-movement Desensitization and Reprocessing therapy (Shapiro et al., 2018). The aim of such therapies is often to restore a sense of safety and connection with the body as the initial stage in the healing process through the use of certain physical actions or movements (Van der Kolk, 2015; Perry & Szalavitz, 2017). Sensorimotor Psychotherapy in particular sees trauma as having disrupted an individual’s relationship with their body causing a sense of mistrust in the autonomic reactions that occur in response to a perceived threat. Therapy begins with acknowledging the “wisdom” of these responses before developing somatic resources to support regulation (Ogden et al., 2006).

Specific models have also been developed with this focus in relation to paediatric clients including Building Underdeveloped Sensorimotor Systems (BUSS) model (Lloyd, 2020), Sensory Motor Arousal and Regulation Treatment (SMART) programme (Warner et al., 2012) and the SAFE PLACE intervention (May-Benson & Sawyer, 2015). All of these programmes focus on a perceived disruption in sensorimotor development, regulation and attachment, resulting from early trauma. They take a “bottom-up” approach to the treatment of trauma viewing sensorimotor development as foundational to higher level skills such as regulation and cognitive skills. Each programme draws sensory integration theory and actively utilise movement experiences as a central part of the therapeutic process. To more fully support the use of such treatments there is a need for further knowledge of this assumed disruption and why it is these approaches appear to be having greater success perhaps than more traditional cognitive based therapies with this client group. This study aims to gain further insight into these underlying assumptions and the nature of the sensorimotor impact of trauma.

## Purpose of this Review

The purpose of this scoping review is to consider what evidence there is for the sensorimotor impact of childhood trauma. This scoping review seeks to broaden the focus beyond that of previous reviews that narrow the focus to sensory modulation and to consider the impact of childhood trauma on sensory based motor function including motor planning, motor skills and postural control. A preliminary search of CINAHL plus, the Cochrane Database of Systematic Reviews and JBI Evidence Synthesis was conducted and no current or underway systematic reviews or scoping reviews on the topic were identified.

This review considers the question - What is known from existing literature about the impact of childhood trauma on sensory processing and connected motor planning and skills?

## Methods

### Search Strategy

The scoping review process followed the JBI methodology for scoping reviews (Peters et al., 2020). An initial limited search of CINAHL plus, Proquest and Scopus was undertaken to identify articles on the topic and inform the search strategy through index terms used and also confirm the databases to be used. It was decided only studies published in English between 2006 and 2022 should be included. A relatively long time period was allowed due to the expected limited range of published evidence on this topic and the absence of previous reviews on this specific area. However as understanding of the impacts of psychological trauma has significantly increased in recent years the period was capped and the initial search proved this to be a sufficient period as all potentially relevant studies were within this period. The search was limited to English language studies due to the resources available for this study not extending to the time and cost of translation. Articles were included if they discussed children or adolescents aged between 0 and 21 years old. Exclusion criteria included co-morbid conditions such as foetal alcohol syndrome and traumatic brain injury.

The primary search was completed by one researcher (RM) using CINAHL plus, Proquest, Scopus, APA PsycINFO and Ethos to identify both published and unpublishes studies such as conference presentations and theses. A complete list of search terms used can be found in Table 1. An additional search was also completed using google scholar to identify grey literature and ensure a more complete picture of what is currently known. The reference lists of all included sources of evidence were screened for additional studies.

**Table 1:** Search terms

Category	Key words
Motor	Motor coordination OR praxis OR motor planning OR motor development
Sensory	Sensory processing OR sensory integration OR sensorimotor OR sensory motor
Trauma	Trauma OR abuse OR neglect OR PTSD OR maltreatment
Age group	Children OR Adolescents OR Youth

The search returned 566 articles for review, with 524 retained for review following removal of duplicates. The articles were initially screened for inclusion against the inclusion criteria based on title and abstract independently by two reviewers with any differences resolved by a third reviewer. Articles were excluded if there was not mention of all three components of trauma, sensory processing and motor skills within the title or abstract. Seven sources were retained for full review and assessed in detail against the inclusion criteria by two reviewers. Six studies were found to meet the criteria. All studies were included, regardless of study quality, due to the sparsity of literature on the topic and to ensure as comprehensive a review as possible.

**Fig. 1:**
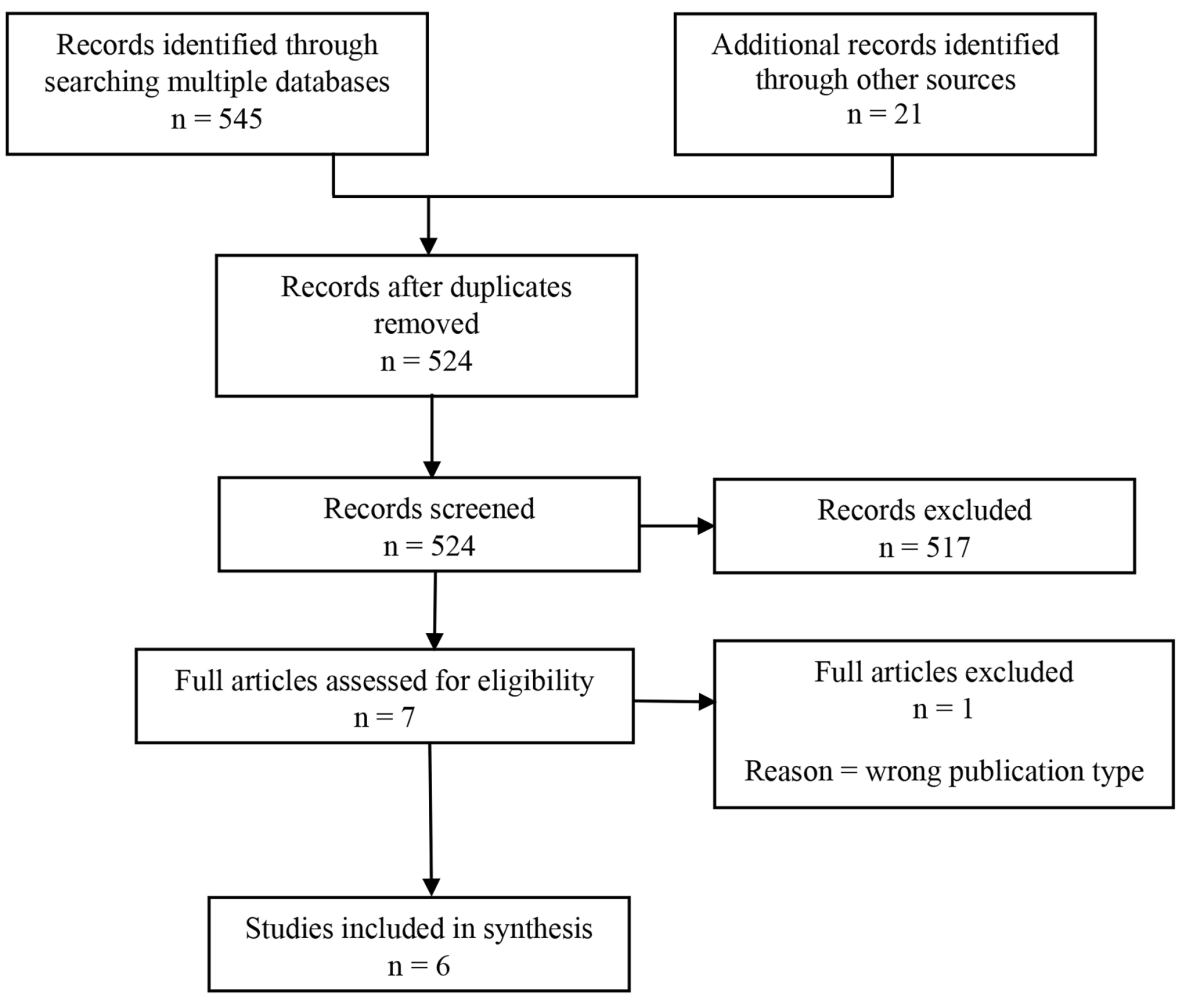
PRISMA chart

### Data Extraction and Analysis

Two authors reviewed the articles to extract the study data independently and conferred to reach an agreement. Information was extracted about authors, year of publication, study location, context, participants, methodology, motor difficulties, sensory processing difficulties discussed and connection to life stage.

## Results

**Table 2:** Data extraction summary table

Citation details	Country	Context	Participants (age, gender, number)	Methodology	Motor difficulties	Sensory processing concerns	Connection to life stage
Elbrecht and Antcliff (2015)	Australia	Clinic based therapy – clay therapy. Description of intervention and observed difficulties.	Children 2 and over.	Practice review and discussion.	Fine motor skills. Balance. Bilateral hand use.	Interoception. Tactile perception/ haptic perception. Vestibular processing.	Pre-verbal trauma primarily.
Finn et al., (2017)	USA	Out-patient clinic. Evaluation of SMART^1^ programme intervention and observed difficulties	N = 1 7-year-old boy	Single case study.	Gross motor skills. Hand-eye coordination.	Hyper-responsivity to visual and tactile input. Proprioception - regulation of speed and force. Proprioceptive and deep pressure seeking.	Pre-verbal trauma.
Ryan, K., Lane, S. J., & Powers, D. (2017)	USA	Out-patient setting. Application of Circle Pre-school Programme^2^ model and noted difficulties.	N = 1 4-year-old boy	Single case study	Rigidity in muscle tone. Motor planning/ control.	Poor sensory processing of gustatory, olfactory, auditory and visual input. High need for vestibular and proprioceptive input.	Prenatal trauma and postnatal up until 9 months.
Teicher, M.H. & Samson, J.A. (2016)	USA	Neuro-imaging findings synthesis. MRI and PET. Considers brain structure, function and connectivity.	Children and adults with a history of childhood trauma.	Literature review	Spatial awareness (due to reduced integrity of superior longitudinal fasciculus). Motor planning (due to prefrontal cortex impairment).	Impaired visual-spatial processing. Somatosensory perception. Most significant impact on these following sexual abuse.	Significant periods of impact: Bilateral hippocampal volume 3–6 years. Grey matter volume at 14–16 years.
Cabrera et al., (2020)	USA	Various settings included – no criteria provided. Integration of existing literature.	Children 0–18 years.	Literature review	Motor planning. Balance. Postural control. Visuo-motor integration. Suggested connection to neurogenesis and myelination of the cerebellum.	Visual-spatial processing (due to alterations in the hippocampus and corpus collosum). Hyper-responsivity (due to impaired amygdala functioning).	Significant periods of impact: Grey matter development 0–6 years. Reduction in volume of hippocampus and amygdala dependent on age.
Guerino, M.R., Briel, A.F. & Araújo, M.D.G.R., (2015)	Brazil	Hippotherapy Centre. Discussion of assessment and intervention.	N = 2 18 years and 21 years old. Experience of abuse at 6–7 years old.	Case study	Coordination. Balance. Postural adjustment/ control.	Not specified – high levels of proprioception and vestibular input in intervention.	Abuse at 6–7 years.

^1^Sensory Motor Arousal Regulation Therapy (SMART) is a somatic therapy focused on improving regulation through sensory motor engagement

^2^The Circle Pre-school Programme is based upon the Neurosequential Model of Therapeutics but with increased focused on sensory components of the intervention

### Socio-demographic Characteristics

As can be seen in Table 2 the majority of studies were completed within the USA with one study completed in Brazil and one in Australia. All studies included were from 2015 onwards suggesting this is a relatively recent developing area of understanding within the literature. A range of ages had been considered from two years old up to 21 years old but with all instances of trauma discussed having occurred during earlier developmental periods. Three of the studies focused upon trauma that occurred during the pre-verbal period of development in particular and the majority focused upon trauma that occurred prior to age seven.

### Clinical Settings

Out of the studies focused directly on clinical practice all four related to out-patient settings and three out of four on specific case studies within those settings. The remaining study by Elbrecht and Antcliff (2015) provided a practice review in relation to a specific approach of clay therapy however was included within the review as it provided helpful consideration of areas of difficulty frequently seen within the setting in the context of therapy as well as clinical analysis of the potential reasons for this. All four of these studies had a primary focus on interventions including clay therapy (Elbrecht & Antcliff, 2015), SMART programme (Finn et al., 2017), hippotherapy (Guerino et al., 2015), the Circle Pre-school Programme (Ryan et al., 2017). However, each contained reflections on the rationale for treatment approach, observations, or assessment data that was useful in considering the impact of trauma therefore were included. The two literature reviews (Teicher & Samson, 2016; Cabrera et al., 2020) did not narrow their focus to specific settings instead considering the breadth of available literature in relation to neuroimaging findings (Teicher & Samson, 2016) and the neurological impacts of childhood maltreatment (Cabrera et al., 2020).

### Motor Skills

A number of areas of motor difficulty were identified within the studies in relation to gross and fine motor skills, balance, coordination, spatial awareness, motor planning, postural control and visuo-motor integration suggesting a broad range of motor impacts resulting from trauma. Three of the studies identified impairments in motor coordination (Guerino et al., 2015; Finn et al., 2017; Elbrecht & Antcliff, 2015) which was connected with reduced body scheme impacting on their ability to use their body effectively within the environment. Elbrecht and Antcliff (2015) discussed specific examples in relation to bilateral coordination with a common area of difficulty seen being children only using one hand within an activity as if unaware of their ability to use the other hand.

Both Guerino et al. (2015) and Ryan et al. (2017) describe an increase in muscle tone or rigidity in movements that impacted on successful execution of motor movements and an apparent “clumsiness”. Finn et al. (2017) suggest an impact in relation to the quality of motor execution describing observed difficulties in relation to grading of force and timing of movement. Postural control was identified as an area of difficulty within two of the studies (Guerino et al., 2015; Cabrera et al., 2020) both of which considered a broader age range suggesting this difficulty may be more apparent or perhaps more significant at a later stage of development. Balance as an area of concern was also only mentioned in these two studies.

Certain studies connected the difficulties identified with specific areas of the brain hypothesised to have experienced change due to trauma. Teicher and Samson (2016) connect the impact on motor skills to alterations within the superior longitudinal fasciculus; reducing spatial awareness, and the pre-frontal cortex; reducing motor planning ability. Cabrera et al. (2020) connect the identified difficulties to the cerebellum suggesting they result from impairments in relation to neurogenesis and myelination.

### Connection to Sensory Processing

Overall the studies did not conclusively make the link between motor difficulties observed and underlying sensory processing impairments but did draw some helpful connections and demonstrate the concurrence of sensory processing and motor impairments. Three of the studies (Elbrecht & Antcliff, 2015; Teicher & Samson, 2016; Finn et al., 2017) suggest impaired perception in the tactile system with Elbrecht and Antcliff (2015) highlighting this as a reason for the tactile rich activity used within intervention suggesting that for children who have experience trauma attention is drawn to internal interoceptive sensations at the expense of exteroceptive sensory development. Teicher and Samson (2016) draw a connection with somatosensory processing but in the specific context of sexual abuse seeing this specific impairment as related to the nature of the abuse. Hyper-responsivity to tactile input is identified in relation to the case study discussed by Finn et al. (2017) with a corresponding focus on deep pressure touch within the intervention which is reported to lead to improved body awareness and coordination as a result.

Impairments in visual processing are identified within four of the studies (Finn et al., 2017; Teicher & Samson, 2016; Ryan et al., 2017; Cabrera et al., 2020) with all of these studies making some connection between this area of difficulty and motor skills. Visual-spatial processing alongside visuomotor are identified as areas that impact on observed clumsiness and difficulty navigating the environment effectively (Cabrera et al., 2020; Teicher & Samson, 2016; Ryan et al., 2017). Two studies suggest an initial difficulty with hyper-responsivity to visual input (Finn et al., 2017; Ryan et al., 2017) suggesting that perhaps the difficulties with visual motor planning are secondary to difficulties with regulation of sensory input.

While the study by Guerino et al. (2015) does not mention any assessment or observation of specific sensory difficulties prior to intervention the need to develop sensory processing is implied in the described intervention plan which the authors highlight is designed to vestibular and proprioceptive processing in the context of identified postural and motor coordination difficulties. Impairments in other sensory systems, auditory, gustatory and olfactory, are also identified by Ryan et al. (2017) however with no apparent connection to motor difficulties.

Three of the studies suggest the neurological basis for the sensory impairments (Cabrera et al., 2020; Ryan et al., 2017; Teicher & Samson, 2016) identifying specific areas of the brain thought to be impacted by the experience of trauma. Teicher and Samson (2016) specifically draw attention to the sensory cortex with their literature review finding multiple studies that note impairment in this region of the brain. Cabrera et al. (2020) and Ryan et al. (2017) both suggest a concurrent impact on areas of the brain relating to regulation of sensory input; the amygdala and hypothalamus, and motor skills; the hippocampus, corpus collosum and cerebellum.

## Discussion and Implications

This review sought to consider the connection between childhood trauma, sensory processing and motor skills, an area which is yet to be fully explored. The link between childhood trauma and sensory modulation is receiving increasing attention within the literature, however as yet the link between experience of childhood trauma, sensory processing and motor skills has not been fully explored. The impact of impaired sensory processing on motor skills has long been acknowledged within the field of sensory integration however the connection in the context of childhood trauma needs further attention.

While this review provides some emerging evidence the studies in this area are small scale and lower quality therefore very much preliminary in nature and inconclusive, however may provide helpful initial insights to guide future research and further practice development. Only six studies were identified that discussed all three factors of concern: childhood trauma, sensory processing and motor skills, and while these articles considered all three factors overall explicit connections were not made between the sensory impairments and motor impact to any significant level. This link is often apparent in the articles, through factors such as the choice of intervention being designed to stimulate sensory systems integral to motor development specifically the tactile, vestibular, proprioceptive and visual systems, but is not clearly articulated. In addition to this out of the studies included within this review the majority, four out of six, only discuss the impact of trauma in the context of the intervention provided rather than in the development of an initial formulation and therefore provide insufficient contextual data to fully evaluate the connection between identified difficulties.

This review has however highlighted further evidence for some commonly suggested factors in the experience of childhood trauma, one of which is the significance of the age at which trauma occurs. A commonality between the studies that met the inclusion criteria is that they either discussed trauma that occurred at an early age, prior to age seven, or highlighted the significance of this period in relation to the impact. While the literature reviews by Teicher and Samson (2016) and Cabrera et al. (2020) encompass literature across broad age ranges both suggest significant periods of impact on grey matter development prior to age six (Cabrera et al., 2020) and bilateral hippocampal volume between the ages of three to six (Teicher & Samson, 2016) providing a focus on the developmental stage at which the trauma occurs. As the primary periods of sensorimotor development occur prior to age seven trauma that occurs during this period is likely to have a greater impact on the overall developmental process and functioning (Van der Kolk, 2005; May-Benson, 2017).

This impact may be secondary to, or in addition to, the difficulties with modulating sensory input that have received the main focus within literature on trauma. These difficulties with modulation have often been connected with dysregulation of the hypothalamic pituitary (HPA) axis leading to fluctuating arousal states that result in periods of extreme hyper-responsivity to sensory input alternating with periods of shut down to sensory input due to the child’s system becoming overwhelmed (Warner et al., 2012; Lehrner et al., 2016). While there has been suggestion of difficulties with sensory modulation occurring alongside motor planning impairments the link between these and of fluctuating arousal levels on praxis has not been fully explored or supported (May-Benson, 2017; Lane, 2020). Only two of the studies included discuss difficulties with hyper-responsivity to sensory input (Finn et al., 2017; Cabrera et al., 2020), with the remaining studies referring more broadly to sensory processing. However, all these studies identify significant dysregulation in affect likely to be reflective of HPA axis over-activation suggesting that difficulties with regulation of sensory input are likely to be present (Lehrner et al., 2016; Lane, 2020). Whether the connection between trauma and motor skills is direct or indirect this lends further support to the importance of early intervention to support the development of new neuronal models during integral periods (Perry & Szalavitz, 2017; May-Benson, 2017). While the adult brain is thought to retain plasticity and the ability to develop new connections intervention at a later point is likely to face added barriers due to the formation of splinter skills and alternative pathways within the brain that may bring added complexity to the intervention process (Cozolino, 2010; Pfeiffer, 2020).

Two of the sources within this review identified these changes within sensory processing and motor skills as the product of changes within specific structures of the brain related to the processing of sensation at the CNS level including the cerebellum, hippocampus, amygdala, superior longitudinal fasciculus, corpus collosum and prefrontal cortex (Cabrera et al., 2020; Teicher & Samson, 2016). One of the main structures highlighted is the cerebellum, an area of the brain that is highly important in motor control and in supporting the process of translating CNS processing and integration of sensation into praxis (Bear, 2020; Bundy & Lane, 2020). In connection with this a number of the sources included in the review discussed the impact within specific sensory including the visual, vestibular, proprioceptive and tactile systems (Elbrecht & Antcliff, 2015; Finn et al., 2017; Ryan et al., 2017; Teicher & Samson, 2016; Cabrera et al., 2020); systems centrally involved in sensory integration and praxis (Bundy & Lane, 2020). This reflects the dual barriers to effective sensory integration suggested by Harricharan et al. (2021) resulting from both perception of sensory input and as well as top-down integration of the inputs within the brain.

There is reason therefore to consider that motor planning difficulties identified within this client group may have a specific aetiology and requires intervention a specific approach different to that used with children who have not experienced trauma. Use of approaches that solely focus upon regulation of arousal, and improving modulation of sensory input, or on development of motor planning are likely to be more limited in their effectiveness and not fully address the functional difficulties experienced. Therapists working with this client group need to ensure both a comprehensive assessment of sensory processing and motor planning as well as intervention with the potential to support development of effective integration and functional performance.

## Future Research

There is a need for studies that evaluate the impact of childhood trauma upon both sensory processing and motor development through the use of reliable and valid assessment measures in relation to all three areas. Current knowledge is insufficient to inform evidence-based practice within this area and requires more conclusive studies. There is growing interest in the use of sensorimotor based interventions for trauma suggesting the benefits of bottom up approaches that target physiological responses prior to more cognitive based traditional interventions. However, the foundation for an effective intervention process needs to be a thorough assessment not only to ensure this is the most appropriate approach but also so that it can be tailored in the most effective way.

## Limitations

The search was limited to studies published in English which may mean that relevant studies published in other languages may have been missed. Known comorbidities were also excluded, such as foetal alcohol syndrome, which may have eliminated potentially relevant articles that also consider childhood trauma however this may have impacted on the clarity of the results. Of the studies included only four were empirical and all of those focused upon very small sample sizes, in most one case study, which while it allowed for more detailed discussion of individual presentation does not allow for frequency of occurrence of the difficulties discussed within this client group. The remaining two studies were literature reviews however both provided a synthesis rather than systematic review of the literature and therefore would be considered lower quality evidence. A further limitation that should be considered is the impact of the search terms and whether these could have been broadened to obtain further studies. The use of the word “sensory” was trialled in the early stages of the review however returned too high a number of irrelevant studies due to the breadth of applications of this word when used without a connected word such as processing. Additional terms in relation to trauma may have been beneficial however such as “adverse experiences”, “adverse childhood experiences” and “post-traumatic stress disorder” rather than solely the abbreviation PTSD.

## Conclusion

This scoping review focused on considering what is known about the connection between childhood trauma, sensory processing and motor skills. A small number of studies were identified that suggested an implied connection between trauma and both sensory processing and motor development, but with none making the link between all three explicit. Through synthesising the findings of these six studies this review provides an outline of what is currently known but suggests a clear need for more extensive research in this area. The increase in use of sensorimotor based approaches within trauma treatment with both children and adults suggests this is an important area of need but understanding why these approaches may be having greater success than more traditional approaches there is a need to first gain further understanding of the underlying changes and impairments caused by early trauma.
